# Supplementary material for: Tough Polyelectrolyte Hydrogels with Antimicrobial Property via Incorporation of Natural Multivalent Phytic Acid
Source: Polymers (Basel). 2019 Oct 21;11(10):1721. doi: 10.3390/polym11101721 (PMC6835581; doi:10.3390/polym11101721)
Supplement: Supplementary file 1 [file polymers-11-01721-s001.pdf]

Supporting Information for Polymers

Tough Polyelectrolyte Hydrogels with Antimicrobial Property via Incorporation of Natural Multivalent Phytic Acid

Hoang Linh Bui [1], *Chun-Jen Huang* [1, 2, 3,*]
1. Department of Biomedical Sciences and Engineering, National Central University, Taoyuan 32023, Taiwan.
2. Department of Chemical and Materials Engineering, National Central University, Taoyuan 32023, Taiwan.
3. R&D Center for Membrane Technology, Chung Yuan Christian University, Taoyuan 32023, Taiwan
* Corresponding authors: Email: cjhuang@ncu.edu.tw (C.-J. H.)

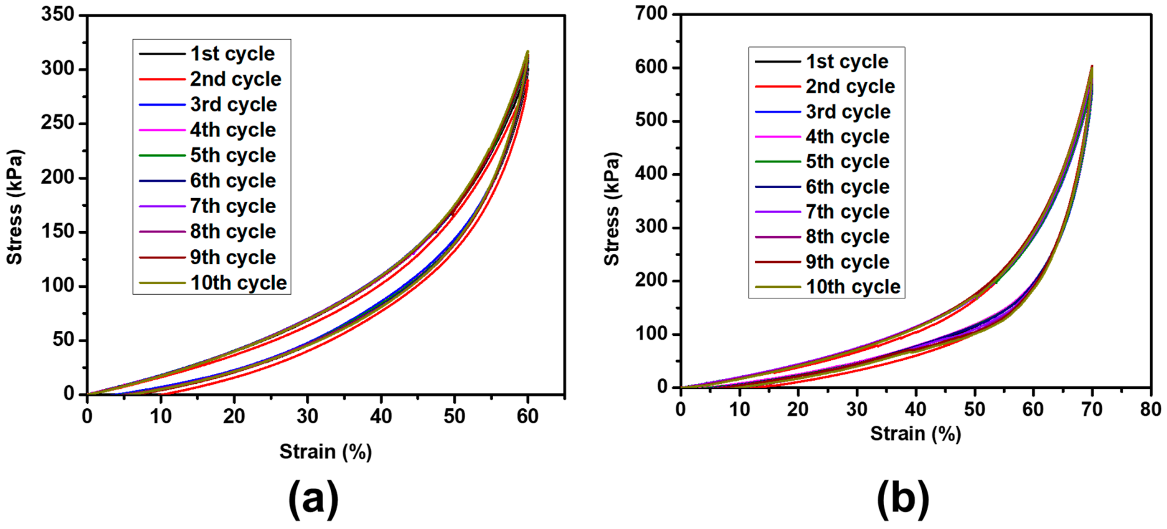

Figure S1. 10 successive loading-unloading cycles of the hydrogel at the deformation of 60% and 70% compressive strain. The compressive test was set at a deformation rate of 6 mm/min at room temperature.

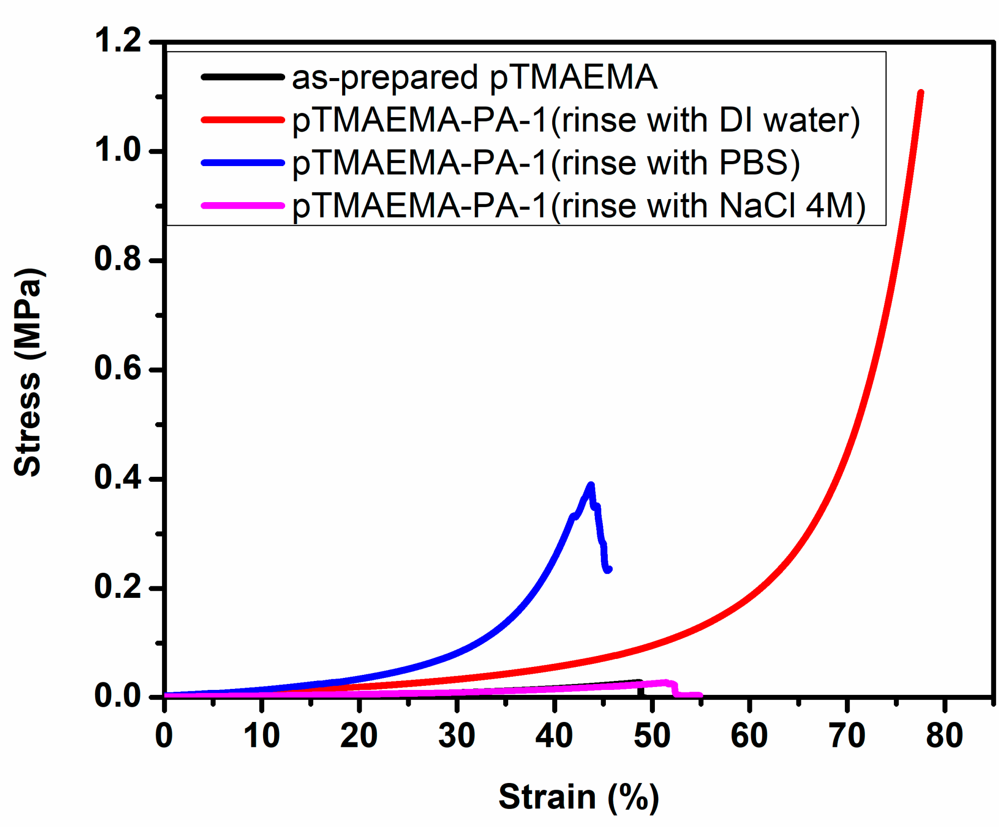

Figure S2. Compressive stress-strain curves of as-prepared pTMAEMA and as-prepare pTMAEMA-PA after rinsing with DI water, PBS 1X and NaCl 4M. The compressive test was set at a deformation rate of 0.5 mm/min at room temperature.

PA release profile

Dried pTMAEMA-PA hydrogels (20 mg) were submerged and shaken in 10 mL PBS (pH 7.4) at 70 rpm to conduct PA released profile in 37 oC. The amount of PA release was measured at 275 nm at room temperature using UV–vis spectrophotometer (model V-600, Jasco). A calibration curve of PA in DI water was established against the absorbance. After a period of time, 1 mL of releasing medium containing TMAEMA-PA hydrogels was withdrawn and replace with 1 mL of fresh PBS medium. The amount of PA releasing was measure in three samples to obtain average value.

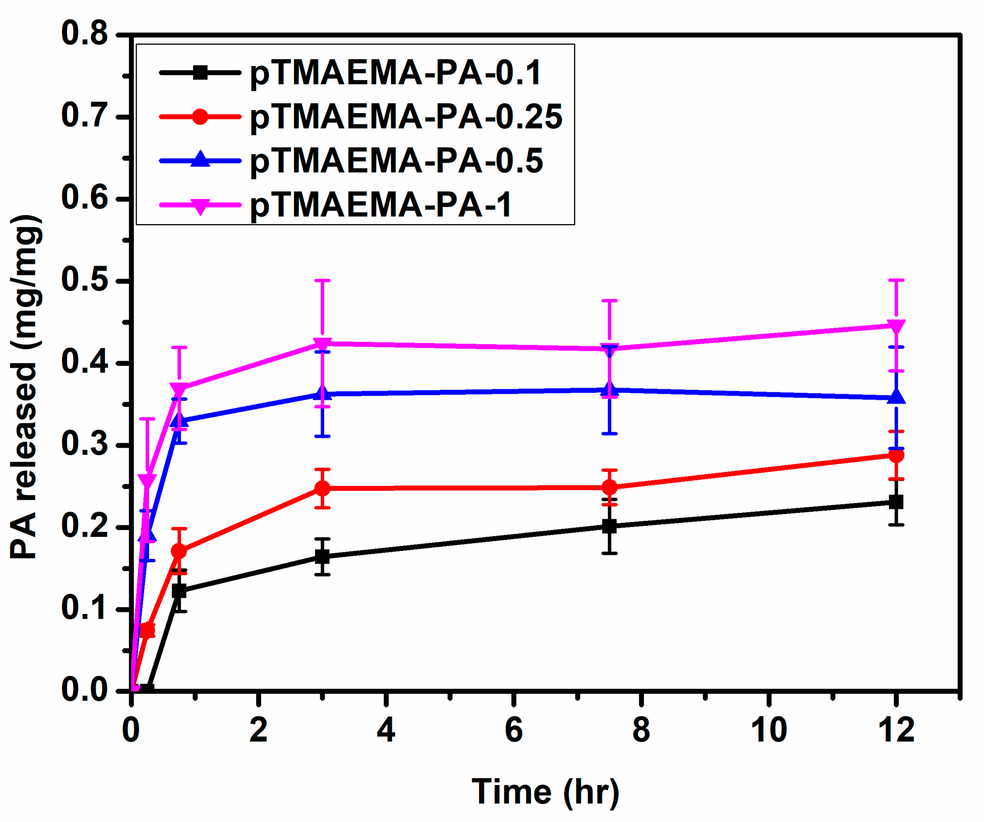

Figure S3. PA release profile of pTMAEMA-PA incubating at 37 °C in PBS solution shaking at 100 rpm.

Extraction cytotoxicity assay

The extraction cytotoxicity assay was based from the ISO 10993-5 standard test method. pTMAEMA and pTMAEMA-PA-0.1 hydrogels were cut into circular discs (7 mm in diameter) and rinse in excess PBS solution for one day prior to the experiment. Then, the hydrogels were sterilized by immersing in 75 % v/v ethanol and again rinsed with sterilized PBS for three times before testing. The extraction medium was prepared by immersing the individual hydrogel in 24-well plates that contained 2 mL of serum-free DMEM medium for 1 and 4 days. NIH/3T3 (mouse fibroblast) cells were cultured in DMEM media (comprised of 10% fetal bovine serum [FBS], 1% penicillin). NIH/3T3 were seeded into 24-well plates at a density of 1×10^4 per well in 1 mL of media for 24 h. The medium was removed and replaced with a serum-free media overnight, then replaced by 500 μL of the prepared extraction media of hydrogels, and the cells were again incubated for 24 hours. The percentage of cell viability was determined using MTT assays. MTT solution (5 mg mL^{-1} in PBS) was diluted by 10 times with serum-free DMEM, and added to each well. Samples were incubated at 37°C for 3 hr. After that, the MTT solution was removed and the precipitated violet crystals were dissolved in 500 μL of DMSO. The absorbance at 550 nm was measured using the ELISA reader (Synergy 2, BioTek, USA). Results were interpreted as average of four samples.

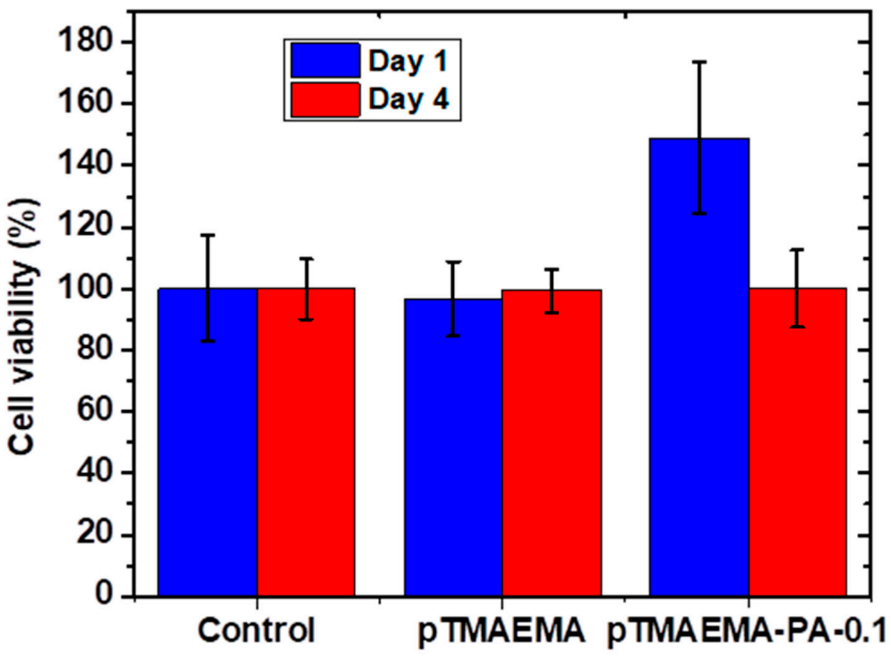

Figure S4. The cell viability of NIH-3T3 fibroblasts co-incubating with pTMAEMA and pTMAEMA-PA-0.1 in the indirect cytotoxicity test.
